# Seasonal Dynamics and Persistency of Endophyte Communities in *Kalidium schrenkianum* Shifts Under Radiation Stress

**DOI:** 10.3389/fmicb.2021.778327

**Published:** 2021-12-16

**Authors:** Jing Zhu, Xiang Sun, Qi-Yong Tang, Zhi-Dong Zhang

**Affiliations:** ^1^Institute of Applied Microbiology, Xinjiang Academy of Agricultural Sciences, Xinjiang Laboratory of Special Environmental Microbiology, Urumqi, China; ^2^School of Life Sciences, Hebei University, Baoding, China

**Keywords:** endophytic fungi, endophytic bacteria, *Kalidium schrenkianum*, halophyte, radiation stress, season

## Abstract

Endophytes are essential components of plant microbiota. Studies have shown that environmental factors and seasonal alternation can change the microbial community composition of plants. However, most studies have mainly emphasized the transitive endophyte communities and seasonal alternation but paid less attention to their persistence through multiple seasons. *Kalidium schrenkianum* is a perennial halophyte growing in an arid habitat with radiation stress (^137^Cs) in northwest China. In this study, *K. schrenkianum* growing under different environmental stresses were selected to investigate the dynamics and persistency of endophytic microbial communities amid seasons in a year. The results showed that Gammaproteobacteria and unassigned Actinobacteria were the most dominant bacterial communities, while the most dominant fungal communities were Dothideomycetes, unassigned Fungi, and Sodariomycetes. The bacterial community diversity in roots was higher than that in aerial tissues, and root communities had higher diversity in summer and autumn. In contrast, the fungal community diversity was higher in aerial tissues comparing to roots, and the highest diversity was in spring. Season was a determinant factor in the microbial community composition in the roots but not in the aerial tissues. RaupCrick index suggested that the bacterial communities were mainly shaped by stochastic processes. Our research investigated the community traits and members with temporal persistency. For example, bacterial taxa *Afipia*, *Delftia*, *Stenotrophomonas*, Xanthomonadaceae_B_OTU_211, and fungal taxa *Neocamarosporium* F_OTU_388, F_OTU_404, F_OTU_445, and unassigned Fungi F_OTU_704, F_OTU_767 showed higher frequencies than predicted in all the four seasons tested with neutral community model. The networks of co-occurrence associations presented in two or more seasons were visualized which suggested potential time-continuous core modules in most communities. In addition, the community dynamics and persistency also showed different patterns by radiation levels. Our findings would enhance our understanding of the microbial community assembly under environmental stress, and be promising to improve the development of integrated concept of core microbiome in future.

## Introduction

Endophytes are microbes living within healthy plant tissues with no adverse effects on plant health (Petrini, 1991). They are essential components of plant microbiota, with high phylogenetic diversity and ecological function. Endophytes assist host plants in adapting to environmental conditions, including biotic and abiotic stresses (Arnold et al., 2003; Rodriguez et al., 2008; Gange et al., 2012). Meanwhile, plants provide physical shade to endophytes against unfavorable conditions, such as dehydration and ultraviolet radiation (Saikkonen et al., 1998; Yao et al., 2020). U’Ren et al. (2012) demonstrated that host plant of different identities selected the endophyte community in various habitats (U’Ren et al., 2012). Season alternations and changes in environmental conditions can alter host plant growth status and the environmental pool of microbe propagules, ultimately shaping the endophyte community (Collado et al., 1999; Gao et al., 2020; Liu and Howell, 2021; Oita et al., 2021). Therefore, season alternation plays a crucial role in determining endophyte communities.

Ionizing radiation comprises high-energy electromagnetic waves or particles that can ionize an atom or a molecule. These ionizing radiations exist in the natural environment and are a form of abiotic stress to organisms. Environmental radiation alters the physiology and morphology of the organisms in radioactive environments and causes negative effects on the ecosystem (Wehrden et al., 2012). Nevertheless, plants, animals, and microbes can survive tolerable or high environmental radiation doses (Shields et al., 1961; Shields and Drouet, 1962; Allred and Beck, 1963; Beatley, 1964; Rickard and Beatley, 1965). Culture-dependent techniques have recovered diverse microbes from radioactive environments (Durrell and Shields, 1960; Zhdanova et al., 2000). Radiation has significant effects on microbial communities. For example, they regulate local soil microbial communities (Zhdanova et al., 2005), gut microbiomes of mammals (Lavrinienko et al., 2018), and endophyte communities of local plants (Zhu et al., 2021). However, how environmental radiation affects the seasonal change of endophytic microbial communities in plants remains unknown.

The core microbiome concept is commonly used in microbiome studies to accommodate community members or features playing important roles in community structure, assembly, maintenance, or function. Although its definition varies due to specific backgrounds and purposes in different studies, the term “core” is committed to depicting the shared features of microbial communities (Shade and Stopnisek, 2019). For example, Turnbaugh et al. (2007) originally proposed that the core microbiome includes all taxa that are common to the microbiomes in all or the vast majority of habitats. Shade and Handelsman (2012) suggested that the core microbiome concept should also include information about community member interactions that are shared among communities. Toju et al. (2018a) emphasized the interactions among community members and defined “core microbiomes” as sets of microorganisms that form cores of interactions that can be used to optimize microbial functions, even if the core species themselves do not have a direct effect on the host. Previous studies have mostly attempted to recover the core microbiome among host individuals or sites (Turnbaugh et al., 2007; Decaens, 2010; Delgado-Baquerizo et al., 2018; Toju et al., 2018b). Only a few focused on temporal persistence among microbial communities over seasons or the annual growth cycle of an economically important plant under agricultural management (Grady et al., 2019; Liu and Howell, 2021). However, the microbial community persistency over various seasons in natural habitats under stress conditions remains unknown.

Endophytes colonize host plants and jointly challenge harsh environmental conditions, including radiation stress (Zhu et al., 2021). However, the annual shift in endophyte communities under radioactive environment is unknown. There are high-radiation habitats caused by Caesium-137 (^137^Cs) accumulation in the arid, saline, and semi-arid desert of northwest China. Seasonal sampling was conducted to collect the aerial tissues and roots of *Kalidium schrenkianum* (Pall.) Moq. (Amaranthaceae halophyte), which is a dominant population of local flora, to investigate the effect of seasonal changes and radiation levels on endophytic microbial communities (bacteria and fungi). This study aimed to reveal: (1) the changes in endophytic communities in response to seasonal alternation; (2) the influence of environmental radiation stress on seasonal dynamics of endophytic communities; (3) the shared features of endophytic communities across the four seasons.

## Materials and Methods

### Study Site and Sampling

Sample collection was conducted in Hoxud County in the Xinjiang Uyghur Autonomous Region of China (91°45′42″E, 40°39′75″N). The area has a semi-arid climate with a mean annual temperature of 12.56°C and mean annual precipitation of 591 mm. Two sites located in a watershed area of seasonal floods were selected for sample collection. One site is a radiation-contaminated region with ^137^Cs accumulation, attributed to natural precipitation and concentration within alluvium of seasonal floods. The other site is 8 km away and not affected by radionuclide (control site). Moreover, the soil had a salt content above 2% on the surface layer (<20 cm deep). Halophyte *K. schrenkianum* was the most dominant plant population in the local habitat, especially at the radionuclide contaminated site.

*Kalidium schrenkianum* plants were collected from two sites with different radioactivity levels in April (spring), July (summer), September (autumn), and December (winter) of 2018. Plants materials were collected using a random sampling approach from a 50 m × 50 m square plot in each site. Five plants per plot were randomly selected (at least 15 m apart) and uprooted. Whole plants were labeled and placed in large autoclaved paper envelopes, then transported to the laboratory in an icebox. The samples were then stored at −80°C until further use. Additionally, five soil samples were collected in September 2018 (autumn) from the surface soil (0–20 cm deep) along the diagonal of the square plot using shovels. A total of 40 samples (2 radiation levels × 4 seasons × 2 tissue types × 5 plant replicates) were used for endophyte analysis. The soil samples from each site were sieved to remove rocks and plant litter, thoroughly mixed, labeled, and packed in cloth bags, and stored at 4°C to transport to the laboratory.

### Analysis of Soil Physical and Chemical Properties

The radioactivity of ^137^Cs in soil samples was analyzed at the Northwest Institute of Nuclear Technology (Xi’an, Shaanxi). One hundred grams of each soil sample were put into an environmental source box (ϕ 45 cm × 25 cm plastic box) and analyzed on an HPGe γ spectrometer (DSPEC-281, ORTEC & DSA-2000, Canberra). Each soil sample was placed 8 cm away from the detector, and the measurement time ranged from 1 to 3 days. The 661 keV peak of ^137^Cs in soil samples was measured and analyzed by net counting, and the radionuclide activity of ^137^Cs in the soil was calculated (Tang et al., 2008). Soil samples from the control site did not show radiation pollution (10–20 Bq/kg), while those from the radionuclide contaminated site showed significantly higher reads (>60 Bq/kg). Also, the physical and chemical indicators of the mixed soil samples from both sites were measured at the Institute of Quality Standards & Testing Technology for Agro-Products, Xinjiang Academy of Agricultural Sciences (Supplementary Table 1).

### DNA Extraction, PCR, and NGS Sequencing

Total DNA was extracted from the aerial parts and roots of *K*. *schrenkianum* separately. Each plant material (20 g) was surface sterilized with 75% (v/v) ethanol for 1 min, 3.25% (w/v) sodium hypochlorite for 3 min, and 75% (v/v) ethanol for 30 s (Guo et al., 2000). Genomic DNA extraction was performed using the cetyltrimethylammonium bromide (CTAB) method (Guo et al., 2000). Briefly, 1 g of the surface-sterilized plant material was freeze-dried in liquid nitrogen, then ground to fine powder using a mortar and pestle. The ground samples were quickly transferred to a tube with 5 ml 2 × CTAB extraction buffer [2% (w/v), 100 mM Tris–HCl, 1.4 M NaCl, 20 mM EDTA, 1.5% (w/v) polyvinyl-pyrrolidone (PVP), 0.5% (w/v) 2-mercaptoethanol; pH 8.0; preheated to 65°C], and then incubated in a 60°C water bath for 30 min with occasional gentle swirling. Next, 500 μl of chloroform: isoamyl alcohol (24:1) was added into each tube and mixed thoroughly to form an emulsion. The mixture was centrifuged at 11,900 *g* for 15 min at room temperature, and the supernatant containing DNA was removed into a fresh 1.5 ml tube and re-extracted twice. Subsequently, 50 μl of 5 M KOAc was added into the supernatant followed by 400 μl of isopropanol and inverted gently to mix. The genomic DNA was precipitated overnight at 4°C and centrifuged at 9,200 *g* for 2 min. The DNA pellet was washed twice with 70% (v/v) ethanol and dried using SpeedVac2 (AES 1010; Savant, Holbrook, NY, United States) for 10 min or until dry. The DNA pellet was resuspended in 100 μl TE buffer (10 mM Tris–HCl, 1 mM EDTA). The DNA concentration and quality were measured using a NanoDrop 1000 Spectrophotometer (Thermo Scientific, Wilmington, NC, United States).

The V5–V7 hypervariable region of the bacterial 16S ribosomal RNA gene was amplified using 799F (AACMGGATTAGATACCCKG) (Chelius and Triplett, 2001) and 1193R (ACGTCATCCCCACCTTCC) primers (Bodenhausen et al., 2013). The fungal internal transcribed spacer region 1 (ITS1 region) of ribosomal RNA was amplified using ITS1F (CTTGGTCATTTAGAGGAAGTAA) (Gardes and Bruns, 1993) and ITS2 (GCTGCGTTCTTCATCGATGC) primers (White et al., 1990). All the PCR reactions were performed in 30 μL reactions containing 15 μL of Phusion^®^ High-Fidelity PCR Master Mix (New England Biolabs, Ipswich, MA, United States); 0.2 μM of forward and reverse primers, and about 10 ng template DNA. Thermal cycling conditions were as follows: initial denaturation at 98°C for 1 min, 30 cycles of denaturation at 98°C for 10 s, annealing at 50°C for 30 s, and elongation at 72°C for 30 s, then a final extension at 72°C for 5 min. The PCR products were mixed with an equal volume of 1× loading buffer (containing SYB green) and separated *via* 2% agarose gel electrophoresis. The expected bands were cut from the gel and purified using GeneJETTM Gel Extraction Kit (Thermo Scientific, Waltham, MA, United States). Sequencing libraries were generated using Ion Plus Fragment Library Kit 48 rxns (Thermo Scientific, Waltham, MA, United States), according to the manufacturer’s instructions. The library quality was assessed on the Qubit@ 2.0 Fluorometer (Thermo Scientific, Waltham, MA, United States). Finally, the library was sequenced on an Ion S5TM XL platform.

### Bioinformatic Analysis

NGS sequencing yielded 79 samples for bacterial dataset and 78 for fungal dataset, as one sample failed to acquire sufficient reads of bacteria and two samples failed for fungi. Raw reads from the bacterial and fungal dataset were demultiplexed and quality filtered using QIIME software (Version1.7.0). Reads with a quality score <20 and those lacking complete barcode and primers were excluded from further analysis. Chimeric sequences were removed using USEARCH software. Subsequently, DADA2 workflow (Callahan et al., 2016) was used to remove singletons and doubletons. Both bacterial and fungal datasets were dereplicated to generate the amplicon sequence variants (ASVs).

The bacterial and fungal taxa were determined using the pipeline described by Zhu et al. (2021). The bacterial and fungal ASVs were first identified using Naïve Bayes approach with a minimum of 75 bootstrap calls following DADA2 workflow (Callahan et al., 2016) against SILVA version 132 (Quast et al., 2013) and UNITE general FASTA release for Fungi version 8.0 (Nilsson et al., 2019). The ASVs which were not assigned to genus (for bacteria) or species (for fungi) level were clustered into different operational taxonomic units (OTUs) based on 97% similarity. One random sequence was selected from each OTU in bacterial or fungal to assign taxonomy with SILVA or UNITE references, respectively. After assigning the taxonomy, all ASVs or OTUs that were assigned to non-bacterial, Cyanobacteria phylum, or Rickettsiales order in the bacterial dataset and to non-fungal in the fungal dataset were removed. The bacterial ASVs or OTUs were then agglomerated at the genus level, while the fungal ASVs or OTUs were agglomerated at the species level, with identical assignments using “phyloseq” package (Callahan et al., 2016).

The ASV and OTU sequences have been deposited in the GenBank of National Center for Biotechnology Information under the accession numbers MZ911967-MZ912492 (bacterial 16S sequences) and MZ919361-MZ919966 (fungal ITS1 sequences). Also, the raw sequences have been deposited in the Sequence Read Archive of NCBI under BioProject PRJNA757585 (for bacterial data) and PRJNA757584 (for fungal data).

Reviewer link:

PRJNA757585
https://dataview.ncbi.nlm.nih.gov/object/PRJNA757585?reviewer=etp8sv9to4q0cie9dldjc40tgd
PRJNA757584
https://dataview.ncbi.nlm.nih.gov/object/PRJNA757584?reviewer=3o9ugraugup7lrg9io6chhgtr5

### Data Analysis

Taxon agglomeration yielded a bacterial and fungal dataset of 2,234,413 and 3,272,864 reads, respectively. Subsequently, singletons and doubletons were filtered from the datasets, and taxa with <0.001 relative abundance were removed to reduce the noise. This resulted in a bacterial dataset of 565 taxa from 79 samples and a fungal dataset of 606 taxa from 78 samples. The bacterial and fungal datasets were then rarefied to 7,500 and 7,400 reads, respectively; the minimum value of read sums among all samples of the dataset. Finally, a bacterial dataset comprising 564 taxa and 79 samples and a fungal dataset containing 606 taxa and 78 samples were generated.

Statistical analyses were performed using R version 3.6.1 (R Development Core Team, 2016). Graphs were plotted with R packages “ggplot2” (Wickham, 2016), “grid” (Murrell, 2005), and “gridExtra” (Auguie, 2017). The distance matrices of endophytic community compositions were constructed by calculating dissimilarities using Bray–Curtis method (Faith et al., 1987). Non-metric multidimensional scaling (NMDS) was used to visualize the community composition dissimilarity of endophytic bacteria or fungi among the different seasons or radiation levels using *metaMDS* function in “vegan” package (Oksanen et al., 2016). Analysis of similarities (ANOSIM) was applied to analyze the differences in microbial composition between plant tissues or radiation levels. Permutational multivariate analysis of variance (PerMANOVA) with 999 permutations was implemented with *adonis* in “vegan” package to determine the environmental influence on microbiota composition. Community diversity of endophytic bacteria or fungi was estimated for incidence data with Hill numbers for *q* = 0, 1, 2 (species richness, the exponential of Shannon entropy, and the inverse of Simpson concentration) using the “iNEXT” package (Hsieh et al., 2016).

The neutral community model (NCM) and Raup-Crick index (RCI) were used to evaluate the stochasticity in the assembly of communities (Sloan et al., 2006; Chase et al., 2011). Bacterial and fungal datasets were conducted for NCM calculation and demonstration using R scripts (Burns et al., 2016) and (Chen et al., 2019). The parameter Nm in the model estimates dispersal between communities. The parameter *R*^2^ represents the overall fit to the neutral model (Sloan et al., 2006). Calculation of 95% confidence intervals around all fitting statistics was done by bootstrapping with 1000 bootstrap replicates. In addition, taxa not fit to neutral model prediction were identified, as higher occupancy or abundance than expected represented plant selection or dispersal limitation (endemic), respectively (Shade and Stopnisek, 2019). RCI was calculated using the R scripts of Chase et al. (2011), and stochasticity was recognized from the proportions of community pairs that fell within | RCI| < 0.95 (Gao et al., 2020).

Correlation analysis was applied for bacterial genera, fungal species, and corresponding unassigned OTUs to deduce inter-taxa interactions. The co-occurrences between endophytic bacterial and fungal taxa in roots or aerial tissues were inspected within each treatment, namely two radiation levels and four seasons. Spearman’s *rho* statistic was used to estimate correlation with function *cor.test* in “stats” package (R Development Core Team, 2016). The frequencies of co-occurrence association (with | *rho*| > 0.8, *p* < 0.01) presented within four seasons were recorded to reveal the association persistency among seasons. If a co-occurrence association between certain taxa presented in two or more seasons, it was kept in a combined network for visualization. The combined co-occurrence networks were visualized with “igraph” package (Csardi and Nepusz, 2006). The structural dissimilarities between networks were calculated according to Schieber et al. (2017). The distance matrices were ordinated with NMDS and plotted using *metaMDS* function in “vegan” package as described above. Network characteristics were determined using functions in “igraph” package.

## Results

### Taxonomical Composition

**FIGURE 1:**
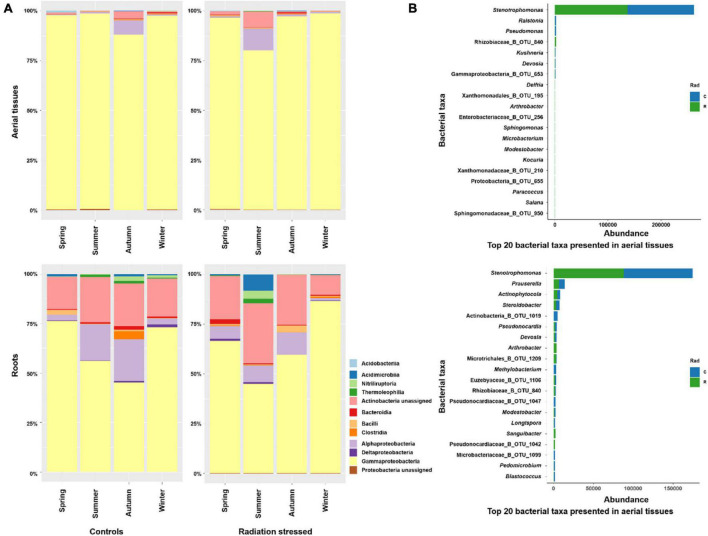
Taxonomical composition of endophytic bacteria in *Kalidium schrenkianum*. **(A)** Relative abundance of top 12 bacterial phyla presented in aerial tissues and roots over different seasons at two sampling sites. **(B)** The abundance of top 20 bacterial genera or OTUs in aerial tissues (upper) and roots (bottom). The blue bars indicate bacterial abundances in the control site, whereas green bars indicate abundances in radiation stressed site.

The bacterial phylum Gammaproteobacteria predominated the bacterial community in aerial tissues of *K*. *schrenkianum* in control and radiation stressed sites, followed by Alphaproteobacteria and unassigned Actinobacteria (Figure 1A). The aerial bacterial communities showed mild seasonal dynamics at the phylum level, that notable proportions of Alphaproteobacteria and unassigned Actinobacteria were observed in the control and radiation stressed sites, during autumn and summer, respectively. Similarly, the dominant phylum in the root community were Gammaproteobacteria, unassigned Actinobacteria, and Alphaproteobacteria. Like aerial communities, there was a seasonal burst of certain phyla in the control site in autumn, and the radiation stressed site in summer, including Acidimicrobiia, Nitriliruptoria, and Thermoleophilia. In the control site, their presence lasted through winter until next spring. At the bacterial genus level, *Stenotrophomonas* predominated aerial and root communities, followed by *Ralstonia* and *Pseudomonas* in aerial tissues and *Prauserella* and *Actinophytocola* in the roots (Figure 1B).

**FIGURE 2:**
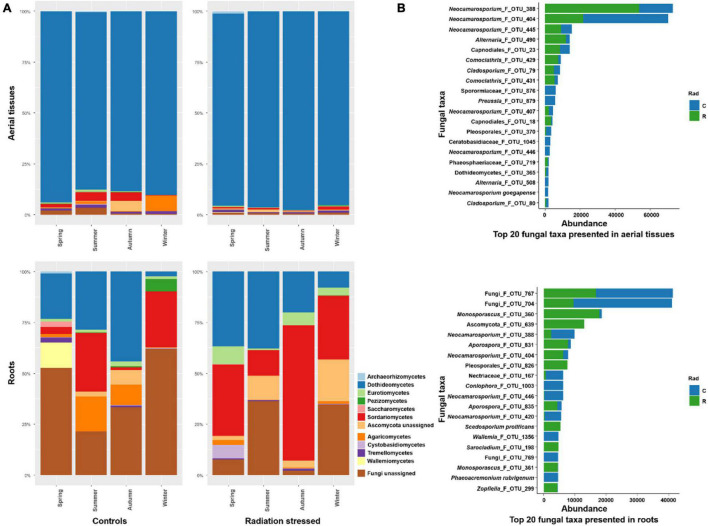
Taxonomical composition of endophytic fungi on *Kalidium schrenkianum*. **(A)** Relative abundance of top 12 fungal phyla presented in aerial tissues and roots over various seasons at two sampling sites. **(B)** The abundance of top 20 fungal species or OTUs in the aerial tissues (upper) and roots (bottom). Blue bars indicate fungal abundances in the control site, and green bars indicate abundances in the radiation stressed site.

The fungal communities showed accordant composition in the aerial tissues and roots at the phylum level, both predominated by Dothideomycetes (Figure 2A). There were more non-Dothideomycetes fungi in the control site than in the radiation stressed site. Notably, unassigned Fungi predominated fungal communities in the roots at both sites, followed by Dothideomycetes and Sodariomycetes. At the fungal species level, *Neocamarosporium* spp. (F_OTU_388 and F_OTU_404) dominated the aerial fungal communities and showed dramatic colonization preference to sites (Figure 2B). The dominant taxa in the root communities were Fungi_F_OTU_767 and Fungi_F_OTU_704, which were more in the control site than the radiation stressed site. The next abundant taxa were *Monosporascus*_F_OTU_360 and Ascomycota_F_OTU_639, mostly inhabiting the radiation stressed site. Intriguingly, the two dominant taxa in the aerial community, *Neocamarosporium*_F_OTU_388 and *Neocamarosporium*_F_OTU_404 were also abundant in the roots, implying that the taxa are not tissue-specific. Notably, Fungi_F_OTU_767 and Fungi_F_OTU_704 might be tissue-specific because they were not observed in aerial tissues.

### Diversity

**FIGURE 3:**
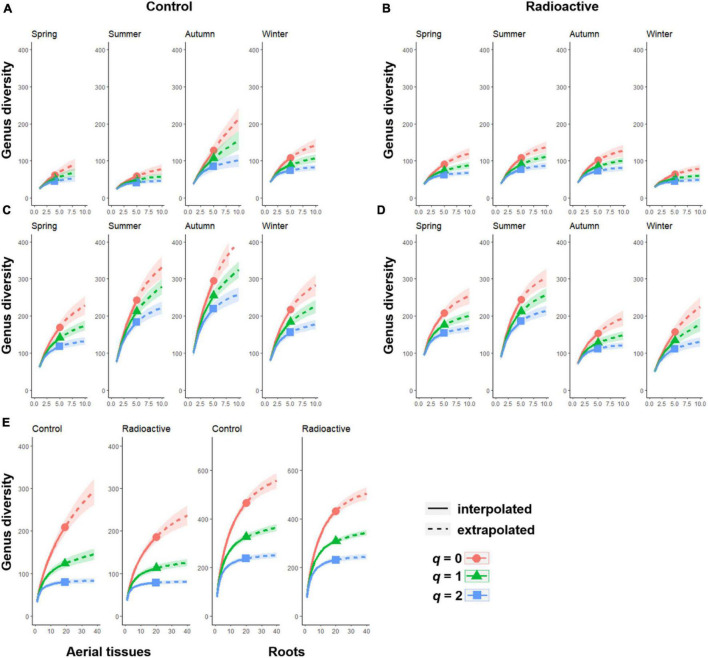
The Hill numbers of order *q indicate the* diversity of endophytic bacteria in each season at the control or radiation stressed sites. The sample size based on rarefactions and extrapolations of four seasons are shown for the bacterial community in aerial tissues **(A)** or in roots **(C)** at the control site, and aerial tissues **(B)** or roots **(D)** at radiation stressed site. The overall rarefactions and extrapolations of different tissues across four seasons are also illustrated **(E)**.

The bacterial community diversity in roots was higher than in aerial tissues at both sites and in all seasons (Figure 3). However, bacterial community diversity in aerial tissues at the control site was relatively low in spring and summer but sharply rose in autumn (Figure 3A). The highest diversity of bacterial communities was recorded in the roots during summer and autumn at the control site (Figure 3C). At the radiation stressed site, the highest bacterial diversity was observed in summer for both aerial and root tissues; however, the diversity of the root community dropped sharply in autumn.

**FIGURE 4:**
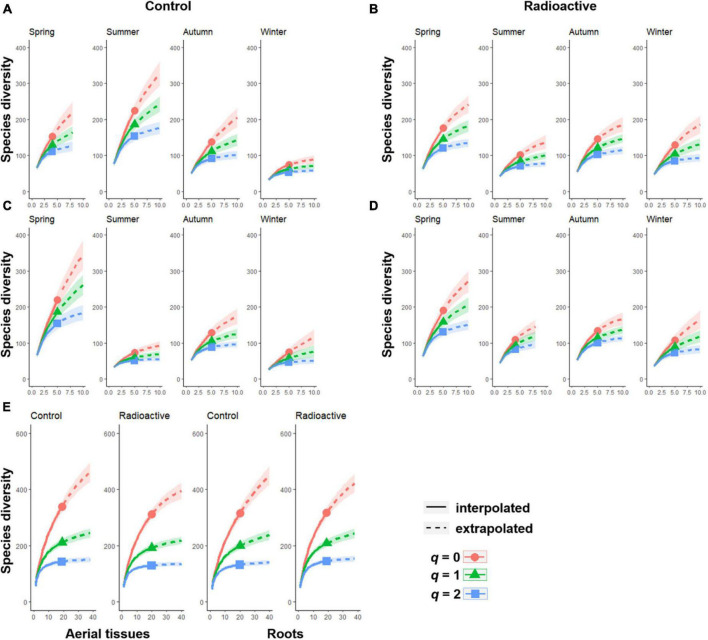
The Hill numbers of order *q indicate the* diversity of endophytic fungi in each season at the control or radiation stressed sites. The sample size based on rarefactions and extrapolations of four seasons are shown for the fungal community in aerial tissues **(A)** or in roots **(C)** at the control site, and aerial tissues **(B)** or roots **(D)** at the radiation stressed site. The overall rarefactions and extrapolations of different tissues across four seasons are also illustrated **(E)**.

The fungal community diversity showed different patterns from that of bacterial communities. In aerial tissues, the diversity was the same or higher than in the roots (Figure 4). The highest fungal diversity in aerial tissues was recorded in summer, while the lowest in winter (Figure 4A). Spring cradled the most diverse fungal communities in the roots at both sites, and aerial tissues at the radiation stressed site. However, fungal diversity levels were relatively low in the other three seasons, with a slight rise in autumn (Figures 4B–D).

### Community Composition

**FIGURE 5:**
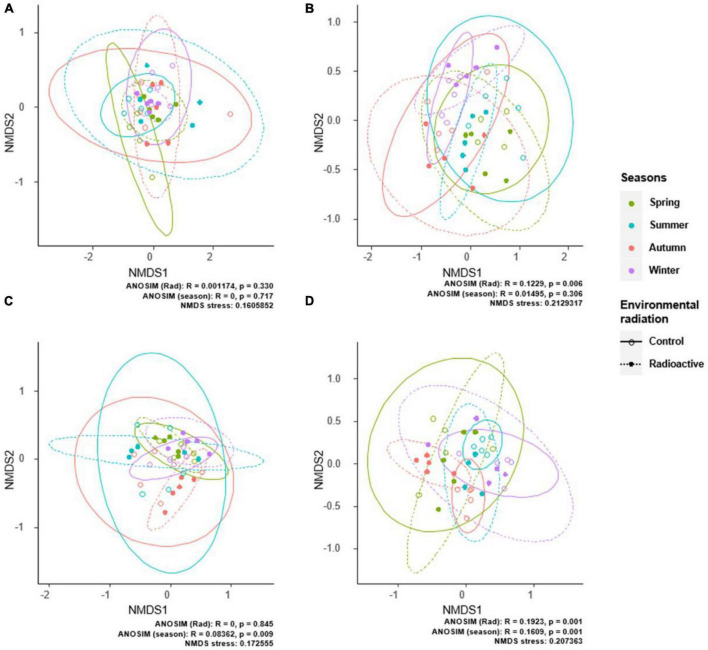
The differences in endophytic microbial communities are demonstrated by non-metric multidimensional scaling ordination. The differences in bacterial communities in aerial tissues **(A)** or roots **(C)**, and fungal communities in aerial tissues **(B)** or roots **(D)** are shown by seasons and sites. Ellipses denote 95% confidence intervals for the centroids of each group. Analysis of similarities analysis indicates significant differences in bacterial and fungal communities between seasons and radiation situations.

Our results demonstrate that radiation and season alternation had no significant effect on the composition of endophytic bacterial communities. PerMANOVA results indicated that radiation stress did not shape bacterial communities with significant effects in aerial tissues (*F*_1_,_37_ = 0.58324, *R*^2^ = 0.01552, *p* = 0.746) and roots (*F*_1_,_37_ = 0.59521, *R*^2^ = 0.01542, *p* = 0.775). Season alternation significantly influenced bacterial community compositions in the roots (*F*_3_,_35_ = 2.2776, *R*^2^ = 0.15952, *p* = 0.011), but not in the aerial tissues (*F*_3_,_35_ = 0.80269, *R*^2^ = 0.06437, *p* = 0.651). ANOSIM results were similar to those obtained by PerMANOVA, reflecting the same patterns of environmental effects on bacterial community compositions. Radiation stress and season alternation did not affect aerial bacterial communities. However, seasonal changes affected bacterial communities in the roots (Figures 5A,C).

Compared to bacterial communities, fungal communities were more sensitive to changes in radiation levels than seasonal alternation. PerMANOVA results suggest that changes in radiation levels affected fungal communities in the aerial tissues and roots (aerial communities: *F*_1_,_37_ = 3.1267, *R*^2^ = 0.07792, *p* = 0.003; root communities: *F*_1_,_37_ = 1.9933, *R*^2^ = 0.05112, *p* = 0.005). Furthermore, seasonal alternation had no significant effect on fungal communities in aerial tissues (*F*_3_,_35_ = 1.1599, *R*^2^ = 0.09043, *p* = 0.253) or root (*F*_3_,_35_ = 1.0634, *R*^2^ = 0.08353, *p* = 0.313). ANOSIM results showed that only the site had significant effect on the fungal community compositions in the aerial tissues (Figures 5B,D). However, the fungal community compositions in the roots were significantly affected by both site and seasonal changes.

### Stochasticity

**FIGURE 6:**
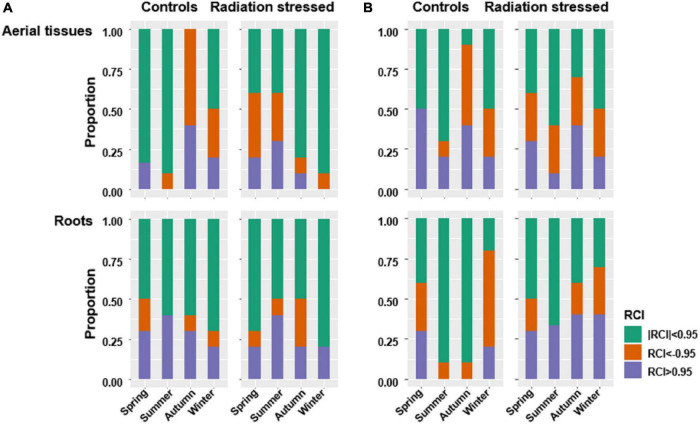
Raup-Crick index was used to detect stochasticity for bacterial communities **(A)** and fungal communities **(B)**. Where | RCI| < 0.95, compositional variance is most likely due to stochasticity.

Raup-Crick index indicated generally high stochasticity in bacterial communities. The stochasticity indicator | RCI| < 0.95 accounted for more than 50% of all pairwise dissimilarities between bacterial communities in different tissue types, sites, and seasons (Figure 6A). Fewer fungal communities exhibited | RCI| < 0.95 larger than 50% (Figure 6B). RCI results suggested seasonal variation of stochasticity in endophyte communities, especially in the roots. The root bacterial communities at both sites showed lower stochasticity in the growing seasons than in winter and spring. In contrast, the highest stochasticity of fungal communities in aerial tissues or roots occurred in summer at both sites. Furthermore, the lowest stochasticity of fungal communities in the roots and aerial tissues occurred in winter and autumn, respectively.

The endophytic communities were tested by a NCM to infer their roles in the community assembly of each taxon. Endophytes with higher or lower frequencies than the prediction of neutral model were identified for each season, and the times not fit to prediction in the four seasons were recorded. *Afipia* and Xanthomonadaceae_B_OTU_211 showed higher frequencies in the aerial tissues than predicted in all the four seasons at control site, while *Delftia* and *Stenotrophomonas* showed higher frequencies in roots at radiation stressed site (Supplementary Table 2). Some fungal taxa, including *Neocamarosporium* spp. (F_OTU_388, F_OTU_404, and F_OTU_445) and Fungi spp. (F_OTU_704 and F_OTU_767) exhibited higher frequencies than predicted in all the four seasons, except in the roots at the radiation stressed site (Supplementary Table 3).

### Network Analysis

**FIGURE 7:**
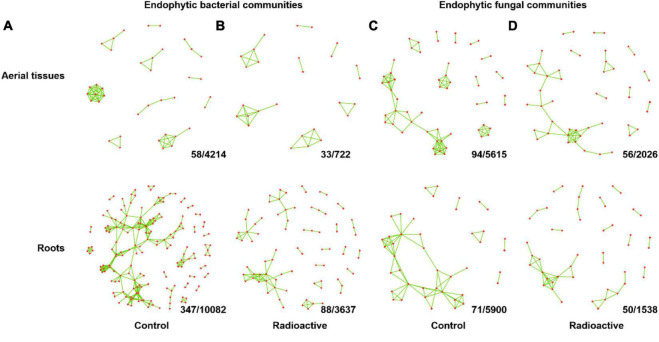
Combined network showing co-occurrence relationships among endophyte communities in two or more seasons. The inter taxa co-occurrences of endophytic bacterial communities in each season at the control site **(A)** or radiation stressed site **(B)** were counted, and co-occurrences relationships presented in only one season were removed. Combined network of endophytic fungal communities at the control site **(C)** and radiation stressed site **(D)** were generated using the same method. Values at the bottom right of each grid indicate the number of co-occurrence relationships presented in no less than two seasons/numbers of co-occurrence relationships presented in at least one season. See Supplementary Table 4 (bacteria) and Supplementary Table 5 (fungi) for counterparts of each co-occurrence relationship in the network graph.

Co-occurrence networks of each endophytic community were built for each season (| *rho*| > 0.8, *p* < 0.01). Divergent distribution in structural similarities between networks was found, especially for fungal communities (Supplementary Figure 1). Combined co-occurrence networks were performed for endophytic communities of aerial tissues and roots at each site, with the co-occurrence association present in two or more seasons (Figure 7). Generally, the endophytes formed sparse networks due to low level of edge densities (Table 1). Bacterial communities in the roots and fungal communities in the aerial tissues at the control site possessed the most connected nodes and edges. The microbial communities in the roots at the control site had the highest centralized betweenness. Nevertheless, the connected endophyte taxa illustrated in network graph were all rare taxa with a relative abundance less than 0.005 (Supplementary Tables 6, 7).

**TABLE 1:** The properties of co-occurrence networks of endophyte communities, which is combined from networks of four seasons.

			Node no.	Edge no.	Edge density	Transitivity	Diameter	Centralized closeness	Centralized eigenvector centrality	Centralized betweenness
Bacterial communities	Control	Aerial tissues	38	58	0.04125178	0.9495413	3	0.00629324	0.833333	0.005641
		Roots	174	347	0.01152747	0.5732379	13	0.00731049	0.9319	0.109489
	Radiation stressed	Aerial tissues	27	33	0.04700855	0.8450704	2	0.00733054	0.877945	0.011716
		Roots	80	88	0.01392405	0.4897959	5	0.00483741	0.916047	0.021553

Fungal communities	Control	Aerial tissues	57	94	0.02944862	0.7521368	7	0.01413701	0.900032	0.103322
		Roots	43	71	0.0393134	0.5551601	5	0.03001756	0.794592	0.18301
	Radiation stressed	Aerial tissues	49	56	0.02380952	0.5833333	9	0.01968354	0.902033	0.141087
		Roots	48	50	0.02216312	0.6428571	5	0.00964648	0.891409	0.031492

## Discussion

The findings of this study agree with those reported in our previous study. Specifically, a higher diversity of bacterial communities in the roots than in aerial tissues and more radiation-sensitive fungal communities than bacterial communities were also observed in our previous study (Zhu et al., 2021). In previous study, bacterial plant microbiome commonly showed similar patterns that higher diversity in roots than in aerial tissues, whereas the fungal diversity is no always higher in aerial tissues (Su et al., 2010; Gdanetz and Trail, 2017). The heterogeneous biochemical environments between roots and aerial tissues, as well as the distinct propagule pools in above ground and underground environments, might cause diversity and composition differences of endophyte communities in roots and aerial tissues (Su et al., 2010; Sun et al., 2012; Fang et al., 2019).

In this study, seasonal alterations and plant growth status jointly shaped the structure of endophyte communities. The abundance of certain bacterial phyla increased significantly in summer at the radiation stressed site and in autumn at the control site in aerial tissues and roots. One possible reason is that the radiation stressed site is located in alluvium, which accumulates radionuclides and nutrients in the region (Supplementary Table 1). Therefore, *K. schrenkianum* at the radiation stressed site grew better and faster in summer, earlier than those at the control site, resulting in a more diverse endophyte community.

Variations in microbial diversity at lower taxonomical levels due to seasonal alterations were more evident in this study. The peak of bacterial diversity was observed in the growth seasons of summer and autumn, while the highest fungal diversity was mostly observed in spring. These results are similar to those reported by previous studies. The diversity of endophytic fungi typically peaks in spring, as previously reported in olive trees in Portugal (Martins et al., 2016). Meanwhile, a study on endophytic bacteria in the shrubs and grasses in Oklahoma, United States, showed that bacterial diversity increases with the growing season of the host plants (Ding and Melcher, 2016). However, studies on the effect of seasonal alternation on endophyte communities in a desert ecosystem are limited.

The community assembly of plant-associated fungi has been studied in agricultural ecosystem. Gao et al. (2020) demonstrated that stochastic forces act on fungal community assembly in the leaves and roots of sorghum during the early stages of development. Similarly, Xiong et al. (2021) reported that in maize, fungal communities are more strongly driven by stochastic processes during the early stages of plant development, while bacterial communities at the later stages of development. Compared to annual crops like sorghum and maize, the community assembly patterns of perennial halophyte *K*. *schrenkianum* are more complicated. For example, the lowest stochasticity of microbial communities in aerial tissues and roots occurred in autumn and winter, respectively (the late stages of annual growth). These results are similar to those reported by previous studies. However, the highest stochasticity of fungal communities was found in summer. Regarding bacterial communities, only those in the roots showed increasing stochasticity from summer to winter. One possible reason is that annual plants assemble microbiota a fresh to begin their growth cycles, while the microbiota assembly of perennial *K*. *schrenkianum* is affected by the endophytes which have already colonized the plant. Therefore, such taxa exert their first-colonizer-priority and interfere with the community assembly of endophytes in the new growth cycle (Wein et al., 2018).

Despite increasing evidence postulating that the stochastic process plays a critical role in microbial community assembly (Zhou and Ning, 2017), microbial communities are not shaped solely by stochastic or deterministic processes (Stegen et al., 2012; Wang et al., 2013; Sun et al., 2020b). Therefore, merely describing a microbial community as stochastic or deterministic does not provide a comprehensive landscape for the microbial community. In this study, we investigated whether an endophyte taxon can fit to neutral model prediction to determine whether stochastic or deterministic processes drove the colonization of a taxon. Our results showed that most taxa exhibited higher occupancy than the prediction (Supplementary Tables 2, 3). Thus, the deterministic process driving community assembly of endophytes was mainly caused by endophyte affinity to host plant rather than dispersal limitation.

Co-occurrence network, which proposes hypothetical interactions between members of a microbial community, has been widely used in ecological or microbiome research. For example, co-occurrence network was used to reveal the heterogeneity of soil microbial community at a microcosm and continental scale (Uksa et al., 2015; Ma et al., 2016), identify antagonistic counterparts between pathogen and biocontrol agents (Blaustein et al., 2017), and uncover host physical or pathological condition-dependent community changes (Baldassano and Bassett, 2016; Biagi et al., 2016). Comparison of networks between communities has been used to explore shared features. Buettner and Noll (2018) found few shared bacterial and archaeal OTUs in their study, indicating that the microbial communities of biogas and sewage-treated plants are distinct in community members and microbial interaction patterns. Sun et al. (2020a) illustrated that certain interaction correlations are shared by co-occurrence networks from different sampling sites in wheat and other related cereal plants. Shade and Handelsman (2012) suggested that core taxa should include community members co-varying within a community and shared across communities. Therefore, the overlapping components of networks should be taken into consideration identifying the core microbiota in future work.

Here, the persistency of inter-taxa co-occurrence associations and endophyte colonization of affinity to host plant were investigated within a year. The results showed that the colonization patterns of endophytes are more persistent, implying that the endophyte colonization patterns are resistant to the disturbance caused by seasonal alternation. Several bacterial and fungal taxa showed persistence throughout the year. However, no co-occurrence association was shared in all four seasons.

In ecological network of a community, the nodes with a high degree and closeness centrality and lowest betweenness centrality in the combined network graph were considered as keystone or hub fungal taxa (Berry and Widder, 2014). Additionally, the edges with high frequencies showed temporal persistency, and would be considered as core interactions (Shade and Handelsman, 2012; Toju et al., 2018a). Interestingly, the bacterial and fungal taxa with a high degree or centrality were mostly rare taxa rather than prevalent ones in this study. Therefore, the dominant endophyte taxa in *K*. *schrenkianum* are probably isolated units of each other in the community.

## Conclusion

This study investigated the seasonal changes of endophyte community associated with halophyte *K. schrenkianum* under environmental radiation stress. According to the results, Gammaproteobacteria and unassigned Actinobacteria were the most dominant bacterial phyla, and Dothideomycetes, unassigned Fungi, and Sodariomycetes were the most dominant fungal phyla. Seasonal alterations affected the diversity of bacterial and fungal communities. Specifically, season was a determinant factor in the composition of microbial communities in the roots but not in the aerial tissues. Stochastic processes mainly shaped the bacterial endophyte communities. Several bacterial and fungal taxa exhibited higher frequencies than predicted by the NCM in all four seasons. Co-occurrence analysis connected rare taxa, not the abundant. This study reveals new insights into understanding the seasonal dynamics and persistency of endophyte community structures under environmental stress and offers potential criteria for determining the core endophytic microbiota.

## Data Availability Statement

The datasets presented in this study can be found in online repositories. The names of the repository/repositories and accession number(s) can be found in the article/Supplementary Material.

## Author Contributions

JZ, XS, and Z-DZ designed the experiments. JZ and Q-YT carried out the experiments. JZ and XS analyzed the experimental results. JZ, XS, and Z-DZ wrote and reviewed the manuscript. All authors contributed to the article and approved the submitted version.

## Conflict of Interest

The authors declare that the research was conducted in the absence of any commercial or financial relationships that could be construed as a potential conflict of interest.

## Publisher’s Note

All claims expressed in this article are solely those of the authors and do not necessarily represent those of their affiliated organizations, or those of the publisher, the editors and the reviewers. Any product that may be evaluated in this article, or claim that may be made by its manufacturer, is not guaranteed or endorsed by the publisher.

## Supplementary Material

Supplementary Figure 1 Network dissimilarity illustrated with non-metric multidimensional scaling ordination. The differences in bacterial networks in aerial tissues **(a)** or roots **(c)**, and fungal networks in aerial tissues **(b)** or roots **(d)** are shown by seasons and sites.

Supplementary Table 1 Radiation levels and geochemical characteristics of sampling sites.

Supplementary Table 2 Bacterial taxa exhibited higher occupancy or higher abundance than neutral model prediction. The times that a taxon violated neutral model prediction among four seasons were indicated in “higher occupancy” or “higher abundance” columns.

Supplementary Table 3 Fungal taxa exhibited higher occupancy or higher abundance than neutral model prediction. The times that a taxon violated neutral model prediction among four seasons were indicated in “higher occupancy” or “higher abundance” columns.

Supplementary Table 4 Frequencies of co-occurrence associations among endophyte bacteria presented in four seasons.

Supplementary Table 5 Frequencies of co-occurrence associations among endophyte fungi presented in four seasons.

Supplementary Table 6 The degree, closeness, eigenvector centrality, and betweenness of bacterial taxa connected in combined networks (Figures 7A,B). The relative abundances (one tissue type at one site in all four seasons) of the taxa were provided.

Supplementary Table 7 The degree, closeness, eigenvector centrality, and betweenness of fungal taxa connected in combined networks (Figures 7C,D). The relative abundances (one tissue type at one site in all four seasons) of the taxa were provided.

## References

[B1] Allred D. M. Beck D. E. (1963). Ecological distribution of some rodents at Nevada atomic test site. *Ecology* 44 211–214. 10.2307/1933209

[B2] Arnold A. E. Mejía L. C. Kyllo D. Rojas E. I. Maynard Z. Robbins N. (2003). Fungal endophytes limit pathogen damage in a tropical tree. *Proc. Natl. Acad. Sci. U.S.A.* 100 15649–15654. 10.1073/pnas.2533483100 14671327 PMC307622

[B3] Auguie B. (2017). *gridExtra**: Miscellaneous Functions for “Grid” Graphics [WWW document].* Available online at: https://CRAN.R-project.org/package=gridExtra (accessed December 4, 2021).

[B4] Baldassano S. N. Bassett D. S. (2016). Topological distortion and reorganized modular structure of gut microbial co-occurrence networks in inflammatory bowel disease. *Sci. Rep.* 6:26087. 10.1038/srep26087 27188829 PMC4870640

[B5] Beatley J. C. (1964). Vascular flora of Nevada Test Site Nye county Nevada. *Am. J. Bot.* 51:687.

[B6] Berry D. Widder S. (2014). Deciphering microbial interactions and detecting keystone species with co-occurrence networks. *Front. Microbiol.* 5:219. 10.3389/fmicb.2014.00219 24904535 PMC4033041

[B7] Biagi E. Franceschi C. Rampelli S. Severgnini M. Ostan R. Turroni S. (2016). Gut microbiota and extreme longevity. *Curr. Biol.* 26 1480–1485. 27185560 10.1016/j.cub.2016.04.016

[B8] Blaustein R. A. Lorca G. L. Meyer J. L. Gonzalez C. F. Teplitski M. (2017). Defining the core citrus leaf- and root-associated microbiota: factors associated with community structure and implications for managing Huanglongbing (citrus greening) disease. *Appl. Environ. Microbiol.* 83:e00210-17. 10.1128/AEM.00210-17 28341678 PMC5440699

[B9] Bodenhausen N. Horton M. W. Bergelson J. (2013). Bacterial communities associated with the leaves and the roots of *Arabidopsis thaliana*. *PLoS One* 8:e56329. 10.1371/journal.pone.0056329 23457551 PMC3574144

[B10] Buettner C. Noll M. (2018). Differences in microbial key players in anaerobic degradation between biogas and sewage treatment plants. *Int. Biodeterior. Biodegrad.* 133 124–132. 10.1016/j.ibiod.2018.06.012

[B11] Burns A. R. Stephens W. Z. Stagaman K. Wong S. Rawls J. F. Guillemin K. (2016). Contribution of neutral processes to the assembly of gut microbial communities in the zebrafish over host development. *ISME J.* 10 655–664. 10.1038/ismej.2015.142 26296066 PMC4817674

[B12] Callahan B. J. Sankaran K. Fukuyama J. A. McMurdie P. J. Holmesa S. P. (2016). Bioconductor workflow for microbiome data analysis: from raw reads to community analyses. *F1000Res*. 5:1492. 10.12688/f1000research.8986.2 27508062 PMC4955027

[B13] Chase J. M. Kraft N. J. B. Smith K. G. Vellend M. Inouye B. D. (2011). Using null models to disentangle variation in community dissimilarity from variation in α−diversity. *Ecosphere* 2:24.

[B14] Chelius M. K. Triplett E. W. (2001). The diversity of archaea and bacteria in association with the roots of Zea mays L. *Microb. Ecol.* 41 252–263. 10.1007/s002480000087 11391463

[B15] Chen W. Ren K. Isabwe A. Chen H. Liu M. Yang J. (2019). Stochastic processes shape microeukaryotic community assembly in a subtropical river across wet and dry seasons. *Microbiome* 7:138. 31640783 10.1186/s40168-019-0749-8 PMC6806580

[B16] Collado J. Platas G. Gonzalez I. Pelaez F. (1999). Geographical and seasonal influences on the distribution of fungal endophytes in *Quercus ilex*. *New Phytol.* 144 525–532. 10.1046/j.1469-8137.1999.00533.x 33862861

[B17] Csardi G. Nepusz T. (2006). The igraph software package for complex network research. *InterJournal Complex Syst.* 1695 1–9. 10.1186/1471-2105-12-455 22115179 PMC3282787

[B18] Decaens T. (2010). Macroecological patterns in soil communities. *Glob. Ecol. Biogeogr.* 19 287–302. 10.1111/j.1466-8238.2009.00517.x PMC3891896 24443643

[B19] Delgado-Baquerizo M. Oliverio A. M. Brewer T. E. Benavent-Gonzalez A. Eldridge D. J. Bardgett R. D. (2018). A global atlas of the dominant bacteria found in soil. *Science* 359 320–325. 10.1126/science.aap9516 29348236

[B20] Ding T. Melcher U. (2016). Influences of plant species, season and location on leaf endophytic bacterial communities of non-cultivated plants. *PLoS One* 11:e0150895. 10.1371/journal.pone.0150895 26974817 PMC4790846

[B21] Durrell L. W. Shields L. M. (1960). Fungi isolated in culture from soils of the Nevada Test Site. *Mycologia* 52 636–641. 10.1007/s10482-018-1062-4 29516314

[B22] Faith D. P. Minchin P. R. Belbin L. (1987). Compositional dissimilarity as a robust measure of ecological distance. *Vegetatio* 69 57–68. 10.1007/978-94-009-4061-1_6

[B23] Fang K. Miao Y. F. Chen L. Zhou J. Yang Z. P. Dong X. F. (2019). Tissue-specific and geographical variation in endophytic fungi of *Ageratina adenophora* and fungal associations with the environment. *Front. Microbiol.* 10:2919. 10.3389/fmicb.2019.02919 31921082 PMC6930192

[B24] Gange A. C. Eschen R. Wearn J. A. Thawer A. Sutton B. C. (2012). Differential effects of foliar endophytic fungi on insect herbivores attacking a herbaceous plant. *Oecologia* 168 1023–1031. 10.1007/s00442-011-2151-5 21989607

[B25] Gao C. Montoya L. Xu L. Madera M. Hollingsworth J. Purdom E. (2020). Fungal community assembly in drought-stressed sorghum shows stochasticity, selection, and universal ecological dynamics. *Nat. Commun.* 11:34. 10.1038/s41467-019-13913-9 31911594 PMC6946711

[B26] Gardes M. Bruns T. D. (1993). ITS primers with enhanced specificity for basidiomycetes - application to the identification of mycorrhizae and rusts. *Mol. Ecol.* 2 113–118. 10.1111/j.1365-294x.1993.tb00005.x 8180733

[B27] Gdanetz K. Trail F. (2017). The wheat microbiome under four management strategies, and potential for endophytes in disease protection. *Phytobiomes* 1 158–168. 10.1094/pbiomes-05-17-0023-r

[B28] Grady K. L. Sorensen J. W. Stopnisek N. Guittar J. Shade A. (2019). Assembly and seasonality of core phyllosphere microbiota on perennial biofuel crops. *Nat. Commun.* 10:4135. 10.1038/s41467-019-11974-4 31515535 PMC6742659

[B29] Guo L. D. Hyde K. D. Liew E. C. Y. (2000). Identification of endophytic fungi from Livistona chinensis based on morphology and rDNA sequences. *New Phytol.* 147 617–630. 10.1046/j.1469-8137.2000.00716.x 33862946

[B30] Hsieh T. C. Ma K. H. Chao A. (2016). iNEXT: an R package for rarefaction and extrapolation of species diversity (Hill numbers). *Methods Ecol. Evol.* 7 1451–1456. 10.1111/2041-210x.12613

[B31] Lavrinienko A. Tukalenko E. Mappes T. Watts P. C. (2018). Skin and gut microbiomes of a wild mammal respond to different environmental cues. *Microbiome* 6:209. 10.1186/s40168-018-0595-0 30477569 PMC6258405

[B32] Liu D. Howell K. (2021). Community succession of the grapevine fungal microbiome in the annual growth cycle. *Environ. Microbiol.* 23 1842–1857. 10.1111/1462-2920.15172 32686214

[B33] Ma B. Wang H. Z. Dsouza M. Lou J. He Y. Dai Z. M. (2016). Geographic patterns of co-occurrence network topological features for soil microbiota at continental scale in eastern China. *ISME J.* 10 1891–1901. 10.1038/ismej.2015.261 26771927 PMC5029158

[B34] Martins F. Pereira J. A. Bota P. Bento A. Baptista P. (2016). Fungal endophyte communities in above- and belowground olive tree organs and the effect of season and geographic location on their structures. *Fungal Ecol.* 20 193–201. 10.1016/j.funeco.2016.01.005

[B35] Murrell P. (2005). *R Graphics.* London: Chapman & Hall.

[B36] Nilsson R. Larsson K.-H. Taylor A. Bengtsson-Palme J. Jeppesen T. Schigel D. (2019). The UNITE database for molecular identification of fungi: handling dark taxa and parallel taxonomic classifications. *Nucleic Acids Res.* 47 D259–D264. 10.1093/nar/gky1022 30371820 PMC6324048

[B37] Oita S. Ibáñez A. Lutzoni F. Miadlikowska J. Geml J. Lewis L. A. (2021). Climate and seasonality drive the richness and composition of tropical fungal endophytes at a landscape scale. *Commun. Biol.* 4:313. 10.1038/s42003-021-01826-7 33750915 PMC7943826

[B38] Oksanen J. Blanchet F. G. Kindt R. Legendre P. Minchin P. R. O’Hara R. B. (2016). *Package ‘vegan’: Community ecology package [WWW document].* Available online at: https://cran.r-project.org/web/packages/vegan/index.html (accessed December 4, 2021).

[B39] Petrini O. (1991). “Fungal endophytes of tree leaves,” in *Microbial Ecology of Leaves*, eds Andrews J. H. Hirano S. S. (New York, NY: Springer-Verlag), 179–197. 10.1007/978-1-4612-3168-4_9

[B40] Quast C. Pruesse E. Yilmaz P. Gerken J. Schweer T. Yarza P. (2013). The SILVA ribosomal RNA gene database project: improved data processing and web-based tools. *Nucleic Acids Res.* 41 D590–D596. 10.1093/nar/gks1219 23193283 PMC3531112

[B41] R Development Core Team (2016). *R: A Language and Environment for Statistical Computing.* Available online at: http://www.R-project.org (accessed December 4, 2021).

[B42] Rickard W. H. Beatley J. C. (1965). Canopy-coverage of the desert shrub vegetation mosaic of the Nevada Test Site. *Ecology* 46 524–529.

[B43] Rodriguez R. J. Henson J. Volkenburgh E. V. Hoy M. Wright L. Beckwith F. (2008). Stress tolerance in plants via habitat-adapted symbiosis. *ISME J.* 2 404–416. 10.1038/ismej.2007.106 18256707

[B44] Saikkonen K. Faeth S. H. Helander M. Sullivan T. J. (1998). Fungal endophytes: a continuum of interactions with host plants. *Annu. Rev. Ecol. Syst.* 29 319–343. 10.1146/annurev.ecolsys.29.1.319

[B45] Schieber T. A. Carpi L. Díaz-Guilera A. Pardalos P. M. Masoller C. Ravetti M. G. (2017). Quantification of network structural dissimilarities. *Nat. Commun.* 8:13928. 10.1038/ncomms13928 28067266 PMC5227707

[B46] Shade A. Handelsman J. (2012). Beyond the Venn diagram: the hunt for a core microbiome. *Environ. Microbiol.* 14 4–12. 10.1111/j.1462-2920.2011.02585.x 22004523

[B47] Shade A. Stopnisek N. (2019). Abundance-occupancy distributions to prioritize plant core microbiome membership. *Curr. Opin. Microbiol.* 49 50–58. 10.1016/j.mib.2019.09.008 31715441

[B48] Shields L. M. Drouet F. (1962). Distribution of terrestrial algae within Nevada Test Site. *Am. J. Bot.* 49 547–554. 10.1002/j.1537-2197.1962.tb14979.x

[B49] Shields L. M. Durrell L. W. Sparrow A. H. (1961). Preliminary-observations on radiosensitivity of algae and fungi from soils of Nevada Test Site. *Ecology* 42 440–441. 10.2307/1932103

[B50] Sloan W. T. Lunn M. Woodcock S. Head I. M. Nee S. Curtis T. P. (2006). Quantifying the roles of immigration and chance in shaping prokaryote community structure. *Environ. Microbiol.* 8 732–740. 10.1111/j.1462-2920.2005.00956.x 16584484

[B51] Stegen J. C. Lin X. Konopka A. E. Fredrickson J. K. (2012). Stochastic and deterministic assembly processes in subsurface microbial communities. *ISME J.* 6 1653–1664. 10.1038/ismej.2012.22 22456445 PMC3498916

[B52] Su Y. Y. Guo L. D. Hyde K. D. (2010). Response of endophytic fungi of Stipa grandis to experimental plant function group removal in Inner Mongolia steppe, China. *Fungal Divers.* 43 93–101. 10.1007/s13225-010-0040-6

[B53] Sun X. Ding Q. Hyde K. D. Guo L. D. (2012). Community structure and preference of endophytic fungi of three woody plants in a mixed forest. *Fungal Ecol.* 5 624–632. 10.1016/j.funeco.2012.04.001

[B54] Sun X. Kosman E. Sharon O. Ezrati S. Sharon A. (2020b). Significant host- and environment-dependent differentiation among highly sporadic fungal endophyte communities in cereal crops-related wild grasses. *Environ. Microbiol.* 22 3357–3374. 10.1111/1462-2920.15107 32483901

[B55] Sun X. Kosman E. Sharon A. (2020a). Stem endophytic mycobiota in wild and domesticated wheat: structural differences and hidden resources for wheat improvement. *J. Fungi* 6:E180. 10.3390/jof6030180 32962177 PMC7557378

[B56] Tang X.-H. Pan X.-B. Wan J.-S. Liu Y.-H. Yang Y.-Q. (2008). Caesium-137 accumulation by halophytes at a radionuclide contaminated site (written in Chinese). *J. Nucl. Agric. Sci.* 22 319–323.

[B57] Toju H. Peay K. G. Yamamichi M. Narisawa K. Hiruma K. Naito K. (2018a). Core microbiomes for sustainable agroecosystems. *Nat. Plants* 4 247–257. 10.1038/s41477-018-0139-4 29725101

[B58] Toju H. Tanabe A. S. Sato H. (2018b). Network hubs in root-associated fungal metacommunities. *Microbiome* 6:116. 10.1186/s40168-018-0497-1 29935536 PMC6015470

[B59] Turnbaugh P. J. Ley R. E. Hamady M. Fraser-Liggett C. M. Knight R. Gordon J. I. (2007). The human microbiome project. *Nature* 449 804–810. 17943116 10.1038/nature06244 PMC3709439

[B60] Uksa M. Schloter M. Endesfelder D. Kublik S. Engel M. Kautz T. (2015). Prokaryotes in subsoil-evidence for a strong spatial separation of different phyla by analysing co-occurrence networks. *Front. Microbiol.* 6:1269. 10.3389/fmicb.2015.01269 26635741 PMC4649028

[B61] U’Ren J. M. Lutzoni F. Miadlikowska J. Laetsch A. D. Arnold A. E. (2012). Host and geographic structure of endophytic and endolichenic fungi at a continental scale. *Am. J. Bot.* 99 898–914. 10.3732/ajb.1100459 22539507

[B62] Wang J. Shen J. Wu Y. Tu C. Soininen J. Stegen J. C. (2013). Phylogenetic beta diversity in bacterial assemblages across ecosystems: deterministic versus stochastic processes. *ISME J.* 7 1310–1321. 10.1038/ismej.2013.30 23446837 PMC3695296

[B63] Wehrden H. V. Fischer J. Brandt P. Wagner V. Kümmerer K. Kuemmerle T. (2012). Consequences of nuclear accidents for biodiversity and ecosystem services. *Conserv. Lett.* 5 81–89. 10.1111/j.1755-263x.2011.00217.x

[B64] Wein T. Dagan T. Fraune S. Bosch T. C. G. Reusch T. B. H. Hülter N. F. (2018). Carrying capacity and colonization dynamics of Curvibacter in the Hydra host habitat. *Front. Microbiol.* 9:443. 10.3389/fmicb.2018.00443 29593687 PMC5861309

[B65] White T. Bruns T. Lee S. Taylor J. (1990). “Amplification and direct sequencing of fungal ribosomal RNA genes for phylogeneics,” in *PCR Protocols: A Guide to Methods and Applications*, eds Innis M. Gelfand D. Sninsky J. White T. (San Diego, CA: Academic), 315–322. 10.1016/b978-0-12-372180-8.50042-1

[B66] Wickham H. (2016). *ggplot2: Elegant Graphics for Data Analysis.* New York, NY: Springer-Verlag.

[B67] Xiong C. Singh B. K. He J.-Z. Han Y.-L. Li P.-P. Wan L.-H. (2021). Plant developmental stage drives the differentiation in ecological role of the maize microbiome. *Microbiome* 9:171. 10.1186/s40168-021-01118-6 34389047 PMC8364065

[B68] Yao X. Chen Z. Wei X. Chen S. White J. Huang X. (2020). A toxic grass Achnatherum inebrians serves as a diversity refuge for the soil fungal community in rangelands of northern China. *Plant Soil* 448 425–438. 10.1007/s11104-020-04440-4

[B69] Zhdanova N. N. Zakharchenko V. A. Haselwandter K. (2005). “Radionuclides and fungal communities,” in *The Fungal Community - Its Organization and Role in the Ecosystem*, eds Dighton J. White J. F. Oudemans P. (Boca Raton, FL: CRC Press), 759–768. 10.1201/9781420027891.ch38

[B70] Zhdanova N. N. Zakharchenko V. A. Vember V. V. Nakonechnaya L. T. (2000). Fungi from Chernobyl: mycobiota of the inner regions of the containment structures of the damaged nuclear reactor. *Mycol. Res.* 104 1421–1426.

[B71] Zhou J. Ning D. (2017). Stochastic community assembly: Does it matter in microbial ecology? *Microbiol. Mol. Biol. Rev.* 81:e00002-17. 10.1128/MMBR.00002-17 29021219 PMC5706748

[B72] Zhu J. Sun X. Zhang Z.-D. Tang Q.-Y. Gu M.-Y. Zhang L.-J. (2021). Effect of ionizing radiation on the bacterial and fungal endophytes of the halophytic plant *Kalidium schrenkianum*. *Microorganisms* 9:1050. 10.3390/microorganisms9051050 34068093 PMC8152737

