# Supplementary figures and images for: Seasonal Dynamics and Persistency of Endophyte Communities in Kalidium schrenkianum Shifts Under Radiation Stress
Source: Front Microbiol. 2021 Dec 16;12:778327. doi: 10.3389/fmicb.2021.778327 (PMC8716815; doi:10.3389/fmicb.2021.778327)

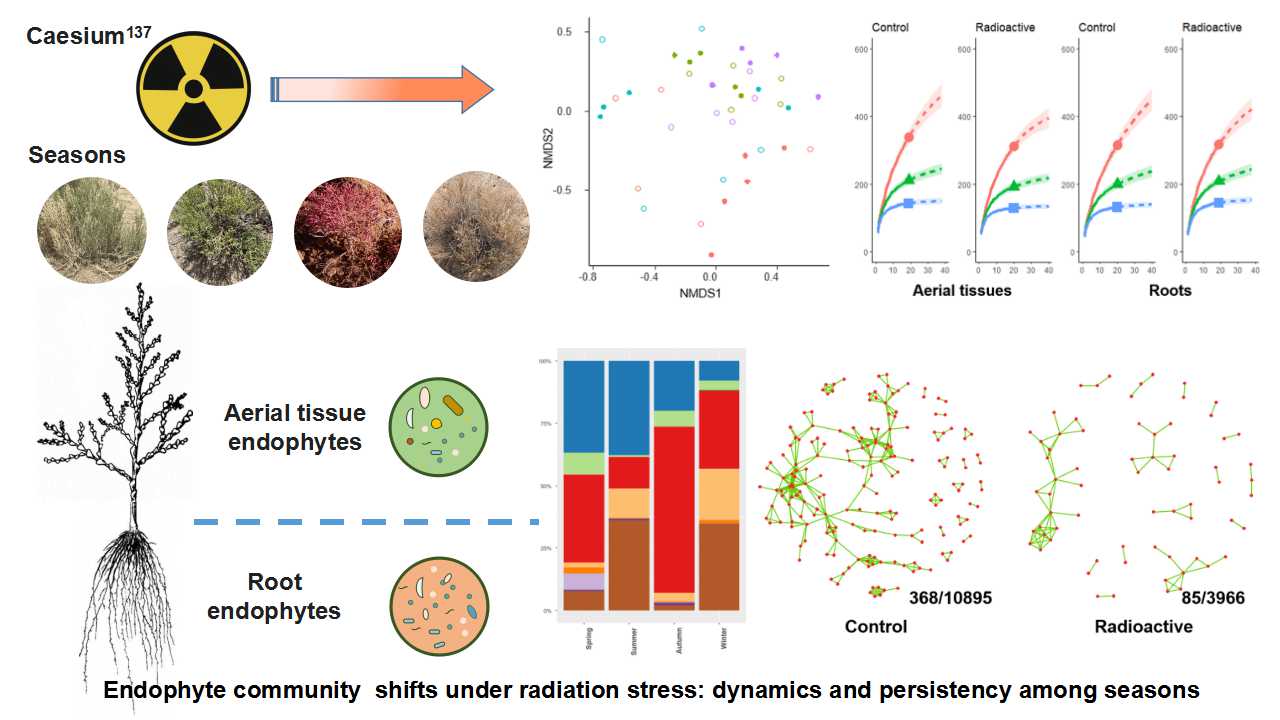

Supplement: Supplementary Figure 1 — Network dissimilarity illustrated with non-metric multidimensional scaling ordination. The differences in bacterial networks in aerial tissues (a) or roots (c), and fungal networks in aerial tissues (b) or roots (d) are shown by seasons and sites. [file Data_Sheet_1.zip › Figure S1.JPEG]

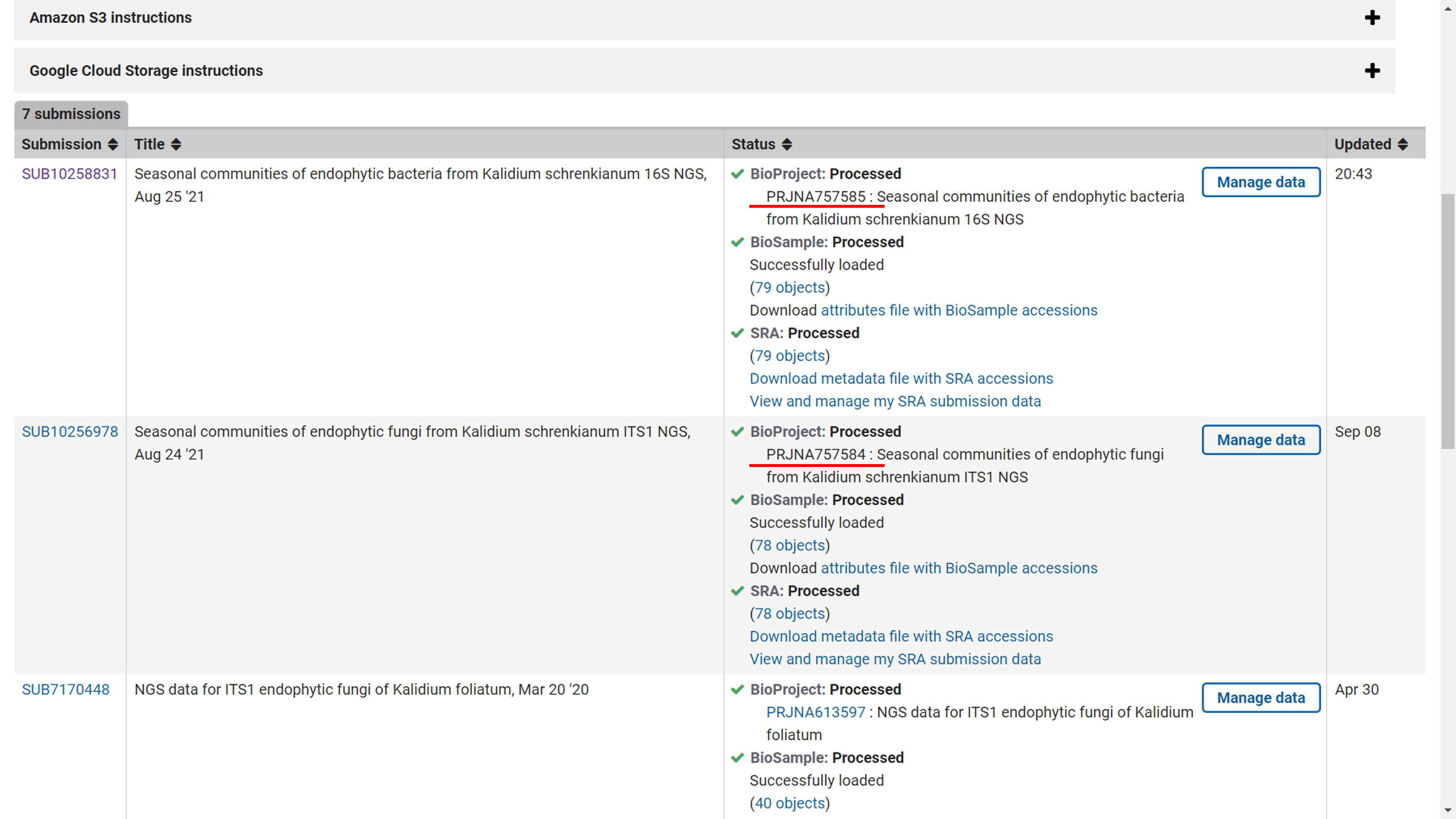

Supplement: Supplementary Figure 1 — Network dissimilarity illustrated with non-metric multidimensional scaling ordination. The differences in bacterial networks in aerial tissues (a) or roots (c), and fungal networks in aerial tissues (b) or roots (d) are shown by seasons and sites. [file Data_Sheet_1.zip › Figure S2.TIF]

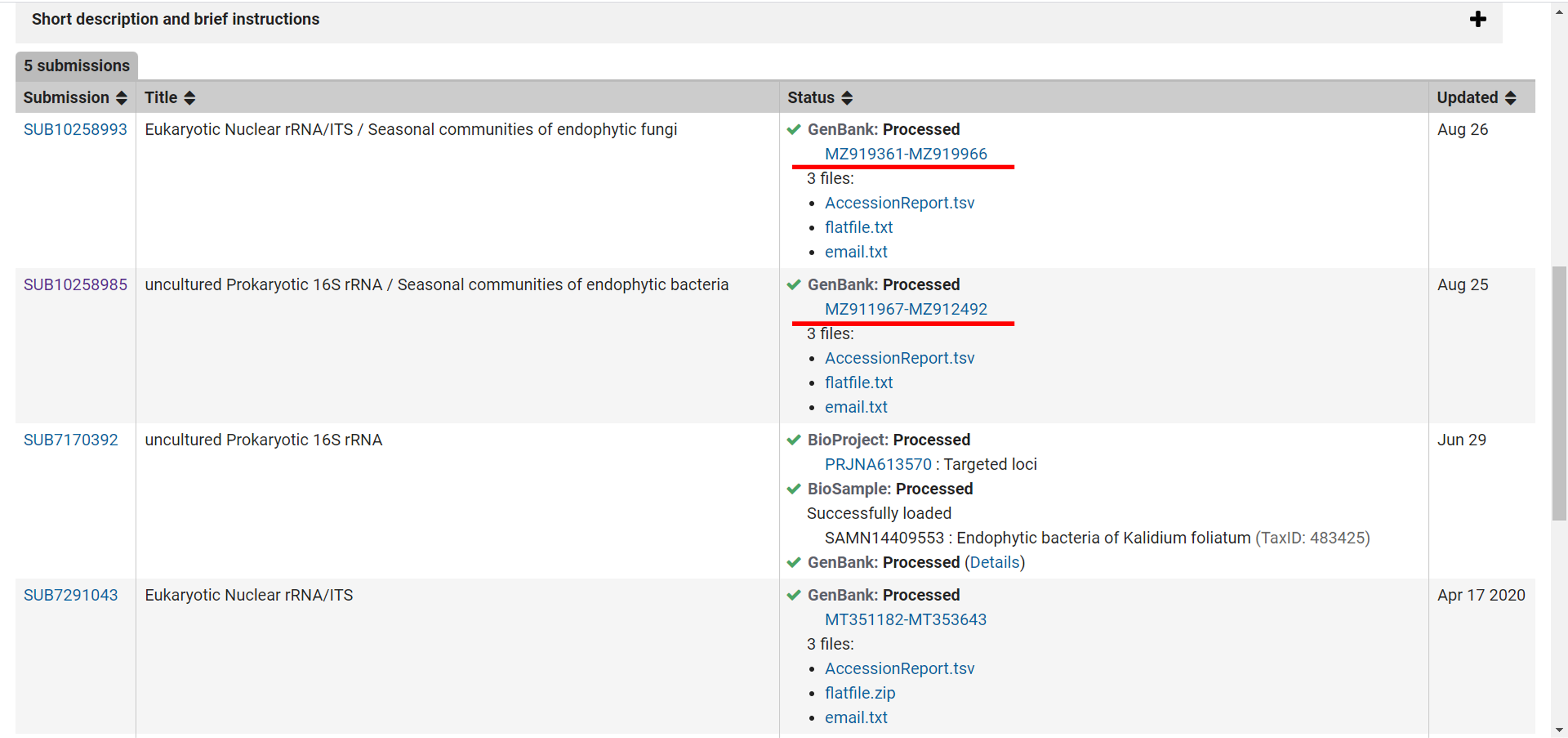

Supplement: Supplementary Figure 1 — Network dissimilarity illustrated with non-metric multidimensional scaling ordination. The differences in bacterial networks in aerial tissues (a) or roots (c), and fungal networks in aerial tissues (b) or roots (d) are shown by seasons and sites. [file Data_Sheet_1.zip › Figure S3.TIF]

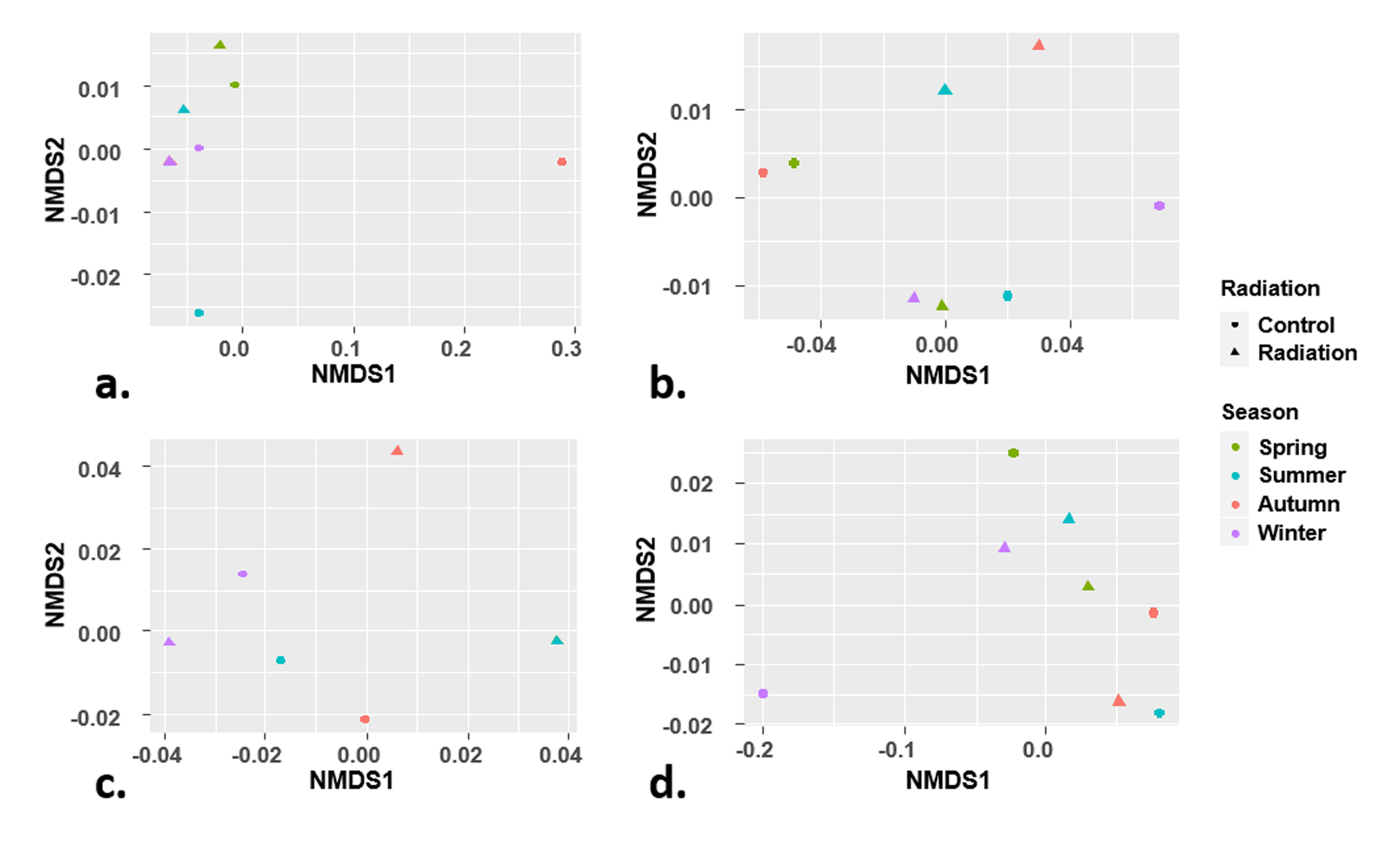

Supplement: Supplementary Figure 1 — Network dissimilarity illustrated with non-metric multidimensional scaling ordination. The differences in bacterial networks in aerial tissues (a) or roots (c), and fungal networks in aerial tissues (b) or roots (d) are shown by seasons and sites. [file Data_Sheet_1.zip › Figure S4.TIF]
